# Geographical Variation in Use of Intensive Care in Denmark: A Nationwide Study

**DOI:** 10.1186/2197-425X-3-S1-A25

**Published:** 2015-10-01

**Authors:** AHS Vestergaard, CF Christiansen, H Nielsen, S Christensen, SP Johnsen

**Affiliations:** Aarhus University Hospital, Department of Clinical Epidemiology, Aarhus N, Denmark; Aarhus University Hospital, Department of Anaesthesiology and Intensive Care, Aarhus N, Denmark

## Introduction

Substantial variation in use of intensive care resources has been reported between countries and within the US, however, data on geographical variation in use within more homogenous tax-supported health care systems are sparse.

## Objectives

The study was aimed at examining whether there is geographical variation in the use of intensive care resources between regions and municipalities in Denmark concerning both admission and use of specific interventions.

## Methods

We conducted a population-based cross-sectional study based on linkage of national medical registries including all Danish residents between 2008 and 2012 using population statistics from Statistics Denmark. Data on intensive care unit (ICU) admission and interventions, including mechanical ventilation, non-invasive ventilation, acute renal replacement therapy and treatment with inotropes/vasopressors, were obtained from the Danish Intensive Care Database. Data on patients' residence at the time of admission were obtained from the Danish National Registry of Patients.

ICU admission rates were age- and gender standardized while proportions of patients receiving different therapies were age-, gender- and comorbidity standardized.

## Results

We identified 125,790 patients, who were admitted to ICUs in Denmark between 2008 and 2012. The overall ICU admission rate in Denmark for the five-year period was 4.6 admissions per 1,000 inhabitants (95% CI, 4.5; 4.6) ranging from 3.9 (95% CI, 3.9; 4.0) to 5.5 admissions per 1,000 inhabitants (95% CI, 5.4; 5.6) in the 5 regions of Denmark and from 2.9 (95% CI, 2.8; 3.1) to 23.5 admissions per 1,000 inhabitants (95% CI, 13.5; 33.5) in the 98 municipalities.

**Figure 1:**
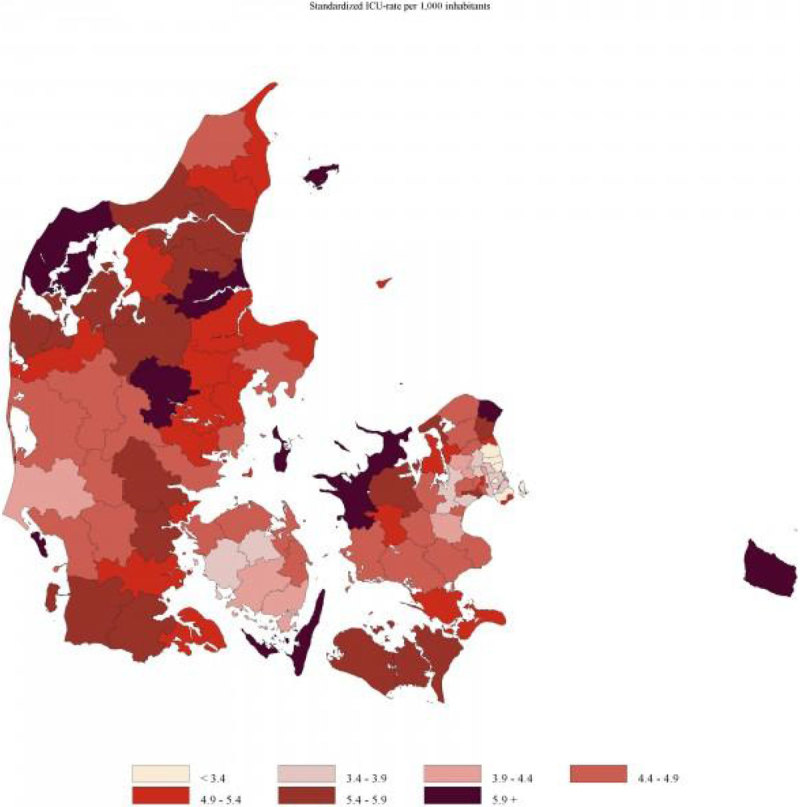
**Std. ICU admission rate per 1,000 inhabitants.**

The standardized proportion of use of interventions among ICU patients also differed across regions and municipalities.

## Conclusions

There is substantial geographical variation in the use of intensive care resources in Denmark both concerning ICU admissions and intensive care interventions.

